# STAT3 mediates C6-ceramide-induced cell death in chronic lymphocytic leukemia

**DOI:** 10.1038/sigtrans.2017.51

**Published:** 2017-10-27

**Authors:** Ushma A Doshi, Jeremy Shaw, Todd E Fox, David F Claxton, Thomas P Loughran, Mark Kester

**Affiliations:** 1 Department of Pharmacology, Penn State College of Medicine, Hershey, PA, USA; 2 Department of Experimental Pathology, University of Virginia School of Medicine, Charlottesville, VA, USA; 3 Department of Pharmacology, University of Virginia School of Medicine, Charlottesville, VA, USA; 4 Division of Hematology and Oncology, Penn State Milton S Hershey Medical Center and College of Medicine, Hershey, PA, USA; 5 Department of Medicine, University of Virginia School of Medicine, Charlottesville, VA, USA; 6 NanoSTAR Institute, University of Virginia, Charlottesville, VA, USA

## Abstract

The pathogenesis of chronic lymphocytic leukemia (CLL) is poorly understood and it remains incurable with current therapies. We have previously shown that nanoliposomal C6-ceramide (CNL) is an effective therapy in an *in vivo* murine model of CLL. However, the key signaling pathways mediating CNL-induced cell death in CLL remains unknown. We hypothesized that CNL targets STAT3, a critical regulator of hematopoietic biology. We observed that CNL treatment reduced phosphorylated STAT3 at both Y705 and S727 residues in CLL cell lines and patient cells. This, in turn, reduced STAT3 transcriptional activity and expression of critical STAT3-dependent survival factors like Mcl-1 and survivin. The effect of CNL on STAT3 was further confirmed *ex vivo* as shown by reduced STAT3 phosphorylation in xenograft tumors obtained from mice treated with CNL. CNL suppressed STAT3 phosphorylation at Y705 and S727 through reduction in BTK activity and MEK1/2 kinase/PKC activities, respectively. Moreover, a synergistic reduction in CLL cell viability was observed on co-treatment with CNL and the BTK inhibitor, ibrutinib. Expression of an oncogenic form of STAT3 conferred partial resistance to CNL, providing confirmation that STAT3 mediates CNL-induced cell death. Taken together, these findings provide the first body of evidence demonstrating ceramide regulation of STAT3 phosphorylation. These results are also the first to demonstrate an effect of ceramide on BTK, a critical kinase mediating the B-cell receptor signaling in CLL cells and suggest a novel and synergistic combination of CNL and BTK inhibitors for CLL treatment.

## Introduction

Chronic lymphocytic leukemia (CLL) is a B-cell malignancy characterized by the clonal expansion and accumulation of neoplastic B lymphocytes expressing CD5, CD19, CD20 and CD23 in the bone marrow, peripheral blood and often the lymph nodes.^1^ Depending on the degree of somatic hypermutation and chromosomal abnormalities, the clinical course of CLL ranges from slow progression to rapid disease progression.^1,2^ The standard treatment regimen of fludarabine, cyclophosphamide and rituximab has an overall response rate of ~90% and complete remission of 72%.^3,4^ Despite these advances in therapeutics, CLL remains incurable resulting in an unmet need for novel therapies.^1^

A large body of evidence has demonstrated that ceramide potentiates signaling cascades leading to cell death. Intracellular delivery of ceramide remains a challenge due to limited solubility and hence cannot be delivered by conventional methods.^5,6^ Our laboratory has developed a nanoliposomal formulation of C6-ceramide (CNL), which is an effective anti-tumorigenic agent *in vivo* in several cancer models.^7–13^ Specifically in CLL, we have demonstrated that CNL selectively targets the Warburg effect by causing downregulation of glyceraldehyde 3-phosphate dehydrogenase and limits tumor growth in an *in vivo* murine model of CLL.^13^ Additionally, inhibiting accumulation of intracellular ceramide prevents fludarabine-induced apoptosis in CLL cells.^14^ PI3K and BTK inhibitors like GS-1101 and ibrutinib, respectively, can overcome B-cell receptor-mediated survival of CLL cells via increasing cellular ceramide while reducing levels of anti-apoptotic glucosylceramide.^15^ Together, these data suggest that ceramide is an effective anti-tumorigenic agent for CLL.

In this study, we sought to identify the molecular basis of CNL-induced cell death in CLL. Signal transducer and activators of transcription (STAT) are latent transcription factors that play a critical role in hematopoietic biology.^16^ In CLL, STAT3 and STAT1 are constitutively phosphorylated at serine-727 (S727) but not tyrosine-705 (Y705).^17^ p-STAT3-S727 has the ability to bind DNA and activate transcription in CLL cells and also associates with complex I of the respiratory chain to impart viability and stress protection to CLL cells.^18,19^ STAT3 inhibitors have shown to sensitize CLL cells to apoptosis, indicating that STAT3 is a promising therapeutic target.^20,21^ Herein, we examine the effects of CNL on the regulation of STAT3 and the role of STAT3 in CNL-induced cell death.

## Methods

### Reagents

Antibodies for STAT3, p-STAT3-S727, p-STAT3-Y705, Mcl-1, Ran, STAT1, p-STAT1-Y701, p-STAT1-S727, STAT2, p-STAT2-Y690, STAT5, Akt-S473, BTK, p-BTK-Y223, p-ERK (T202/Y204), ERK, p-MARCKS (Ser 152/156), MARCKS, survivin, XIAP, cyclin D1, p21 and β-actin were purchased from Cell Signaling Technology Inc (Danvers, MA, USA). The anti-FLAG antibody was purchased from Sigma (St Louis, MO, USA). For western blotting, precasted Nupage electrophoresis gels were purchased from Invitrogen (Carlsbad, CA, USA) and chemiluminescence reagent was obtained from Thermo Scientific (Waltham, MA, USA). STAT3 inhibitor, Stattic; MEK inhibitor, U0126 and PKC inhibitor, Bis-I were purchased from Sigma. BTK inhibitor, ibrutinib, was purchased from MedChem Express (Monmouth Junction, NJ, USA).

### Patient characteristics and preparation of peripheral blood mononuclear cells

All patients met the clinical criteria of CLL and were not on treatment at the time of sample acquisition (Table 1). Peripheral blood specimens from CLL patients were obtained and informed consents signed for sample collection using a protocol approved by the Institutional Review Board of Penn State University Hershey. Peripheral blood mononuclear cells (PBMCs) from CLL patients were chosen for experiments according to the following criteria: CD19+ >80%, CD20+ >80%, CD5+ >90%. These criteria ensured that the PBMCs isolated from CLL patient blood predominantly consisted of leukemic B cells. Buffy coats from normal donors were also obtained from the blood bank of Penn State University Hershey. PBMCs were isolated by Ficoll-Hypaque gradient separation, as described previously.^22^

### Isolation of CD19+ B cells

Whole blood from healthy donors was purchased from Research Blood Components (Brighton, MA, USA) and delivered the following day. PBMCs isolated from the whole blood were sorted using positive selection through CD19 MicroBeads (Miltenyi Biotec, Cambridge, MA, USA). Isolated CD19+ B cells were then lysed in RIPA buffer with phosphatase and protease inhibitors (Thermo Pierce, Waltham, MA, USA and Sigma, respectively).

### Cell culture

Freshly isolated PBMCs and primary CLL patient cells were cultured in RPMI-1640 (Invitrogen) medium supplemented with 10% FBS. JVM-3 cells (DSMZ—German Collection of Microorganisms and Cell Cultures, Germany), a CLL cell line with wild-type p53, were also cultured in this same medium. Mec-2 cells (DSMZ), a CLL cell line with mutated p53 were cultured in Iscove’s MDM media supplemented with 10% FBS. HEK-293FT cells (Invitrogen) were cultured in D-MEM supplemented with 10% FBS and 1× anti-anti antibiotic (Gibco, Waltham, MA) containing 10 k units ml^−1^ of penicillin, 10 k μg ml^−1^ of streptomycin and 25 μg ml^−1^ of Gibco Amphotericin.

### Preparation of nanoliposomal ceramide

C6-ceramide nanoliposomes, ghost nanoliposomes and dihydro-C6-ceramide liposomes were prepared as described by Ryland *et al.*^13^ Ghost nanoliposomes were used as negative control in experiments since they have the exact lipid composition as CNL, except for C6-ceramide.

### Preparation of lipid: BSA complexes

Sphingosine:BSA complexes and sphingosine-1-phosphate (S1P):BSA complexes were prepared as described by Hankins *et al.*^23^

### Cell viability assay

A set of experiments were conducted to determine the toxicity of CNL and Stattic in JVM-3 cells, Mec-2 cells, CLL patient cells and in normal donor PBMCs. Cell viability was assessed by a CellTiter 96 Aq_ueous_ One Solution assay kit (Promega, Madison, WI, USA) following the manufacturer’s instructions. All samples were assayed in triplicate and each experiment was repeated at least three times.

### Cell death assays (flow cytometry for Annexin-V/7AAD)

Apoptosis was determined in JVM-3, Mec-2 and CLL patient cells by flow cytometry with Annexin-V-PE and 7-amino-actinomycin D (BD Pharmingen, San Diego, CA, USA) staining using 5×10^5^ cells per sample.

### Western blot analysis

Western blot analysis was performed on whole cell lysates. Densitometry analysis was performed using ImageJ software.

### shRNA knockdown of STAT3

STAT3 shRNA plasmid clones (Human pTRIPZ vector) were purchased from Open Biosystems (Lafayette, CO, USA) and used to transfect JVM-3 cells. Nucleofection was performed using the Amaxa Nucleofector I device. JVM-3 cells (3×10^6^ cells) were resuspended in 100 μl of Cell line Solution Kit V (Amaxa, Seattle, WA, USA) and nucleofected with 6 μg of shRNA using the Amaxa Nucleofector I device (program X-001). Transfection efficiency by this protocol was ~80% as measured by a comparable GFP plasmid provided by the manufacturer.

### Preparation of pervanadate

An aliquot of 1 mM pervanadate (PV) stock was prepared by adding 10 μl of 100 mM sodium orthovanadate, 50 μl of 0.3% hydrogen peroxide diluted in 20 mM HEPES and 940 μl of water. After 5 min of incubation, some catalase was mixed in the PV stock to remove excess hydrogen peroxide. PV was used within 2 h of preparation.

### Luciferase reporter assay

Cignal reporter assay kit from Qiagen (Frederick, MD, USA) was used for obtaining plasmids for the luciferase reporter assay. JVM-3 cells (2×10^6^ cells) were nucleofected with 4 μg of either reporter construct, negative control construct or positive control construct. Cells were cultured for 24 h post transfection and then treated for 12 h with CNL or ghost liposomes. Dual-Glo luciferase assay system from Promega was used to obtain luciferase luminescence. The assay and quantification was done following the manufacturer’s instructions.

### Lentiviral transduction for STAT3-C expression

Human EF.STAT3C.Ubc.GFP vector from Addgene (Cambridge, MA, USA) was used for expressing STAT3-C in JVM-3 cells.^24^ Human pLOC overexpression vector (Open Biosystems) containing a RFP sequence was used as a negative control. Briefly, viral particles were produced in HEK293-FT cells using the vector, VSVG, tat and DR8.2 plasmids. JVM-3 cells were transduced thrice with the viral media. Cells were grown for 72 h after the last transduction. The STAT3-C overexpression vector has an EGFP sequence as a selectable marker and the transduced cells were sorted for EGFP and grown as a pure population (JVM3-STAT3C cells). FACS was not performed for the control JVM3-RFP cells since a transduction efficiency of 70–80% was obtained. Seventy-two hours after last transduction, cells were collected for experiments. JVM3-STAT3C cells and JVM3-RFP cells were treated with 40 μM CNL or ghost liposomes for 24 h. Cell death was then analyzed by flow cytometry.

### Statistical analysis

All data are expressed as mean±s.e.m. All the graphs represent at least three independent experiments, each replicated in triplicate, unless specified otherwise. Paired Student’s *t*-test (two-tail paired) or two-way analysis of variance test were used to determine the statistical significance and *P*-value of 0.05 or less was considered statistically significant.

Combination indices (CI) for synergism analysis were computed with CompuSyn software. CI<1 indicates synergism, CI=1 indicates additive effect and CI>1 indicates antagonism.

## Results

### STAT3 is a potential therapeutic target in CLL

**Figure 1:**
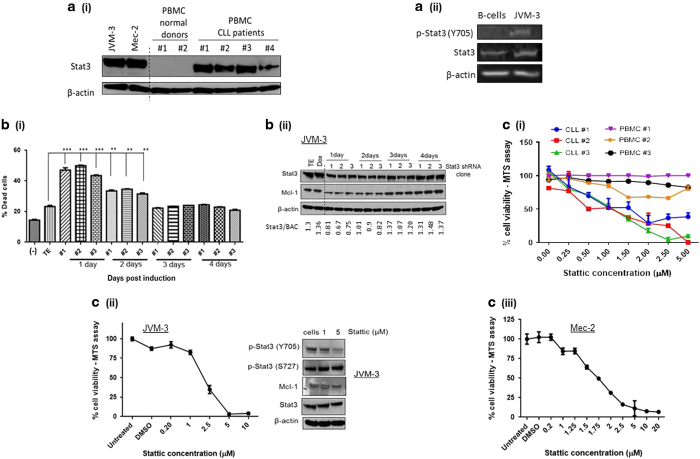
STAT3 is a potential therapeutic target in CLL. (**a**) STAT3 is overexpressed in CLL cell lines and patient cells. (i) JVM-3 cells, Mec-2 cells, PBMCs from two different normal blood donors and PBMCs from four CLL patients were lysed and western blot analysis was performed. The final image was created by grouping different parts of the same film of the same gel as indicated by the black dividing line. (ii) CD19+ B cells were isolated from blood donated by healthy donors and protein levels were compared to JVM-3 cells by western blot analysis. (**b**) Knockdown of STAT3 induces cell death in CLL cells. JVM-3 cells were transfected with several clones of STAT3 shRNA. (i) Flow cytometric analysis was performed to determine % dead cells 24–96 h after doxycycline induction and (ii) western blot analysis done. Cells nucleofected with TE buffer containing no plasmid were used as a control. An aliquot of 1 μg ml^−1^ doxycycline was used to induce the expression of STAT3 shRNA 24 h after nucleofection and doxycycline level was maintained during the assay period. The graph represents two independent experiments. Student’s *t*-test was used for statistical analysis, ***P<0.0001, **P<0.05. The final western blot image was created by grouping different parts of the same film of the same gel as indicated by the black dividing line. (**c**) STAT3 inhibition reduces viability of CLL cell lines and patient cells. (i) PBMCs from three normal donors and three CLL patients, (ii) JVM-3 cells and (iii) Mec-2 cells were treated with Stattic for 24 h and cell viability was determined by the MTS assay. (ii) Western blotting analysis in JVM-3 confirms the effectiveness of Stattic treatment. The graphs represent results from three independent experiments.

Several reports suggest that STAT3 contributes to the pathogenesis of CLL.^18,19,21^ We compared the levels of STAT3 between PBMCs from healthy blood donors and CLL patient cells. STAT3 was overexpressed in both CLL cell lines and patient cells when compared to PBMCs from healthy blood donors (Figure 1a(i)). As shown in Figure 1a(ii), total STAT3 was overexpressed in JVM-3 cells when compared to CD19+ B cells isolated from healthy blood donors, and STAT3 was constitutively active in JVM-3 cells as seen by STAT3 phosphorylation at Y705. We next evaluated if inhibiting STAT3 signaling in CLL cells would induce cell death. Knockdown of STAT3 in JVM-3 cells using an inducible lentiviral STAT3 shRNA significantly increased the number of Annexin-V-positive cells 24 h after doxycycline induction (Figure 1b(i)). An average of 57% knockdown of STAT3 protein was observed 24 h after induction with doxycycline (Figure 1b(ii)). Doxycycline was non-toxic to JVM-3 cells at dosages used. As demonstrated in Figure 1b(i), 24 h after doxycycline induction STAT3 knockdown caused a 108% increase in cell death compared to control cells. As the knockdown weakened at later time points, cell death induction reduced to 42% 48 h after induction, and further reduced as protein levels resumed to normal levels 72 and 96 h post induction (Figure 1b(i) and (ii)). Reduction in STAT3 protein levels also corresponded with a simultaneous reduction in Mcl-1, a protein under the transcriptional control of STAT3 (Figure 1b(ii)).^25^ We also confirmed the role of STAT3 signaling in maintaining CLL cell viability by treatment with Stattic, a small molecule inhibitor of STAT3.^26^ Stattic inhibits STAT3 signaling by selectively inhibiting activation, dimerization and nuclear translocation of STAT3.^26^ Treatment with Stattic for 24 h resulted in a dose-dependent reduction in cell viability of three different CLL patient samples, whereas PMBCs from normal blood donors were relatively resistant to the treatment (Figure 1c(i)). Furthermore, treatment with Stattic resulted in a similar reduction in viability of two different CLL cell lines—*TP53*^*wild-type*^ JVM-3 cells and *TP53*^*mutated*^ Mec-2 cells (Figure 1c(ii) and (iii)). Taken together, these results demonstrate that STAT3 is essential for CLL cell survival.

### CNL suppresses STAT3 phosphorylation

**Figure 2:**
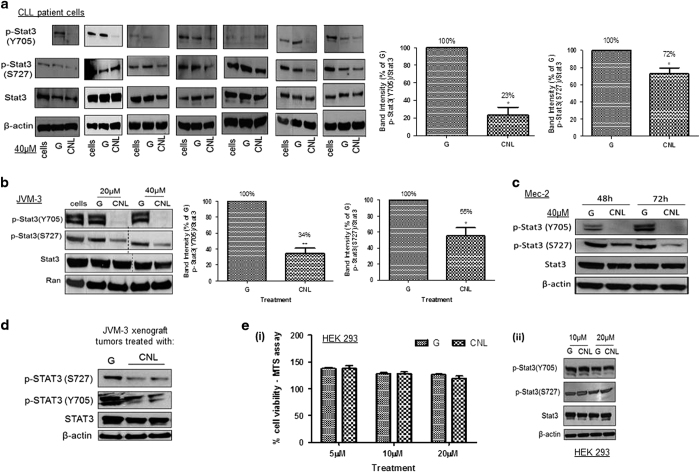
CNL suppresses the phosphorylation of STAT3 at both Y705 and S727 residues. CNL suppresses phosphorylation of STAT3 in (**a**) CLL patient cells; (**b**) JVM-3 cells; (**c**) Mec-2 cells; (**d***) ex vivo* xenograft tumors. Cells were treated with 20 μM and/or 40 μM of ghost nanoliposomes or CNL as indicated in the figure for 24 h (CLL patient cells and JVM-3 cells) or 48 and 72 h (Mec-2 cells). Western blotting analysis was performed. The graphs represent the quantification of western blotting from: (**a**) 7 CLL patient cells; and (**b**) three independent experiments. The final western blot image was created by grouping different parts of the same film of the same gel as indicated by the black dividing line. Statistical analysis was performed using Student’s *t*-test, *P<0.05, **P<0.01. (**d**) JVM-3 xenograft tumors were obtained from a subcutaneous CLL mouse model in Balb/c Nu/nu mice that were injected with ghost nanoliposomes or CNL (from Ryland *et al.*^13^). Western blotting was performed for one tumor treated with ghost nanoliposomes and two CNL-treated tumors obtained from two separate mice. (**e**) CNL does affect cell viability and STAT3 phosphorylation in HEK293 cells. (i) Cell viability of HEK293 cells was determined by MTS assay after 24 h treatment and (ii) western blotting analysis was performed. The results are representative of three independent experiments.

We have previously demonstrated that CNL induces a dose-dependent reduction in CLL cell viability and induces cell death.^13^ To elucidate the molecular mechanism of CNL-induced cell death, we examined the effect of CNL on STAT3 phosphorylation at doses that induced cell death. Cells from seven CLL patients (described in Table 1) were treated with 40 μM CNL or ghost nanoliposomes (no C6-ceramide) for 24 h and STAT3 phosphorylation was evaluated. We observed a reduction in STAT3 phosphorylation at both Y705 and S727 residues in six out of seven patient samples, while total STAT3 levels remained unchanged (Figure 2a). Consistent with these results, JVM-3 cells treated with only CNL (40 μM, 24 h) demonstrated a reduction in STAT3 phosphorylation at Y705 and S727 by 67% and 45%, respectively (Figure 2b), while total STAT3 remained unchanged. We observed similar results on STAT3 phosphorylation in Mec-2 cells treated with CNL (Figure 2c). We have previously shown that CNL displays anti-leukemic activity in a CLL animal model.^13^ We determined STAT3 phosphorylation from representative JVM-3 xenograft tumors from mice injected with ghost nanoliposomes or CNL. Consistent with our *in vitro* data, STAT3 phosphorylation was reduced at Y705 and S727 in tumors from mice injected with CNL (Figure 2d). Thus, CNL reduces STAT3 phosphorylation *in vitro* and *in vivo*. We also tested the effect of CNL on non-transformed HEK293 cells. CNL treatment did not affect the viability of HEK293 cells (Figure 2e(i)), nor did it impact STAT3 phosphorylation (Figure 2e(ii)), thereby demonstrating that this phenomenon is specific to cancer cells.

### Suppression of STAT3 phosphorylation is specific to STAT3 and C6-ceramide

**Figure 3:**
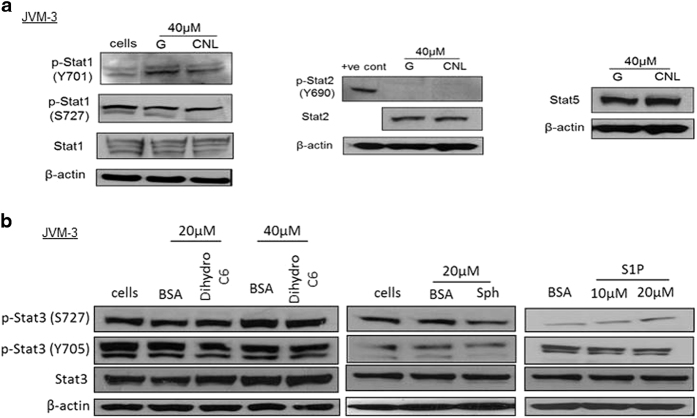
Suppression of STAT3 phosphorylation is specific to STAT3 and C6-ceramide. (**a**) CNL-induced suppression of phosphorylation is specific to STAT3. JVM-3 cells were treated with 40 μM ghost nanoliposomes or CNL for 24 h and western blotting analysis was done. A positive control of STAT2 phosphorylation at Y690 was also used. The images are representative of three independent experiments. (**b**) Only C6-ceramide sphingolipid suppresses STAT3 phosphorylation. JVM-3 cells were treated with dihrdro-C6-ceramide nanoliposomes or BSA:sphingosine complex or BSA:S1P complex for 24 h. Western blotting analysis was performed. The images are representative of three independent experiments.

We next evaluated the specificity of CNL-induced suppression of STAT3 phosphorylation by examining the effect on other STATs and effect of other sphingolipid metabolites on STAT3. We observed that CNL treatment did not significantly impact p-STAT1 (Figure 3a). Basal phosphorylation of STAT2 (Figure 3a) and STAT5 (not shown) was not observed. Total STAT2 and STAT5 remained unchanged after treatment with CNL (Figure 3a). STAT4 was not detected in JVM-3 cells. We next examined if STAT3 dephosphorylation was specific to C6-ceramide sphingolipid. We tested three other sphingolipids: dihydro-C6-ceramide that lacks the double bond in the sphingoid backbone, sphingosine and sphingosine-1-phosphate. None of these sphingolipids significantly changed STAT3 phosphorylation (Figure 3b). We observed an increase in STAT3 phosphorylation at S727 on treatment with sphingosine-1-phosphate. Although this is an interesting finding which fits the current understanding of the ceramide/sphingosine-1-phosphate rheostat and the opposing roles of the two sphingolipids in the promotion/suppression of tumors, however, it is out of the scope of this work. Together, these results prove the specificity of CNL-induced suppression of STAT3 phosphorylation.

### CNL induces necrotic cell death in CLL cells

**Figure 4:**
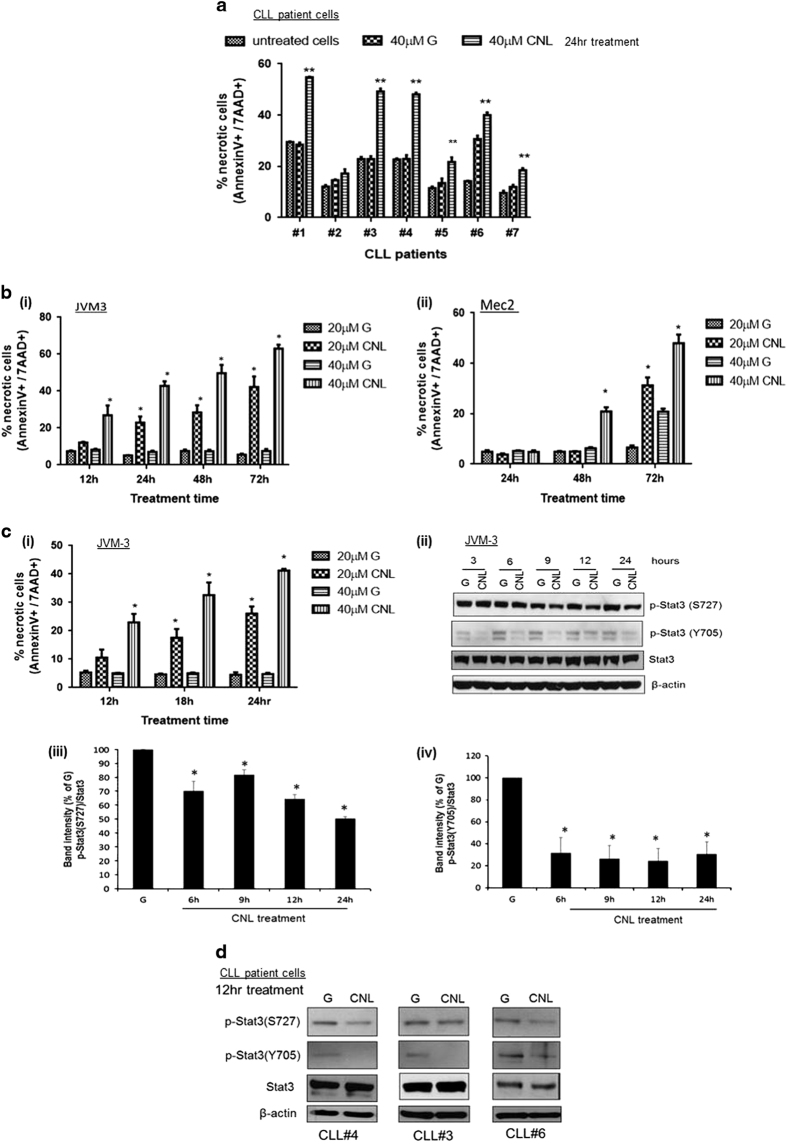
CNL induces necrotic cell death in CLL cells. CNL induces necrotic cell death in (**a**) CLL patient cells and (**b**) CLL cell lines JVM-3 and Mec-2. Cells were treated with 20 μM and/or 40 μM ghost nanoliposomes or CNL for indicated time periods. Flow cytometric analysis using Annexin-V and 7AAD staining was performed to determine the effect on cell death. (**a**) The graph represents the quantification of all seven CLL patient samples. Student’s *t*-test was used to perform statistical analysis **P<0.01. (**b**) The graphs represent the quantification of results from three independent experiments. Two-way ANOVA with Tukey’s multiple comparisons test was used to perform statistical analysis *P<0.01. (**c**) CNL-induced suppression of p-STAT3 precedes induction of cell death (i) JVM-3 cells were treated with ghost nanoliposomes or CNL for indicated time periods and flow cytometric analysis was performed to determine % cell death. The graph is a quantification of three independent experiments. Statistical analysis was done using Student’s *t*-test *P<0.05 (ii) JVM-3 cells were treated with 40 μM ghost nanoliposomes or CNL for indicated time periods and western blotting was performed. (iii) and (iv) Graphical representation of western blotting. The graph is a quantification of three independent experiments. Statistical analysis was done using Student’s *t*-test *P<0.05. (**d**) CNL induces early suppression of p-STAT3 in CLL patient cells. Cells from three CLL patients were treated for 12 h with 40 μM ghost nanoliposomes or CNL and western blotting was done.

We have previously demonstrated using multiple approaches that CNL selectively induces caspase 3/7-independent cell death in CLL cells and cell death resembles a necrotic morphology. No change in caspase activity was observed following treatment with CNL and cell death resembling necrotic morphology was confirmed by phase contrast microscopy.^13^ We confirm these findings by flow cytometric analysis using Annexin-V and a viability dye, 7AAD. Several reports suggest that necrotic cell death is enumerated in the Annexin-V-7AAD double-positive quadrant.^27,28^ We evaluated the effect of CNL on CLL cells obtained from seven patients. Treatment with 40 μM CNL for 24 h induced significant cell death in six out of the seven patients tested (Figure 4a), while ghost nanoliposomes had a minimal effect. Significant cell death was also induced in both *TP53*^*wild-type*^ JVM-3 cells and *TP53*^*mutated*^ Mec-2 cells on CNL treatment (Figure 4b(i) and (ii)). Current literature suggests that CLL patients with p53 pathway dysfunction have poor prognosis due to reduced response to conventional chemotherapies, suggesting an increased resistance to current therapies.^4,29^ As expected, we observed that cell death in *TP53*^*mutated*^ Mec-2 cells was induced after longer treatment with CNL in comparison to *TP53*^*wild-type*^ JVM-3 cells (Figure 4b). Taken together, these results indicate that CNL effectively induces cell death in both *TP53*^*mutated*^ and *TP53*^*wild-type*^ CLL cells.

We next assessed if suppression of STAT3 phosphorylation preceded induction of cell death. Significant cell death in JVM-3 cells was observed 12 h after CNL treatment (Figure 4c(i)). Suppression of STAT3 phosphorylation at Y705 and S727 started as early as 3 h and between 6 and 9 h post treatment, respectively (Figure 4c(ii), (iii) and (iv)). Reduction in STAT3 phosphorylation preceded cell death, suggesting that STAT3 dephosphorylation might potentially mediate cell death. Consistent with this, we also observed suppression of STAT3 phosphorylation in three CLL patient cells after 12 h of treatment with CNL (Figure 4d). Overall, these results demonstrate that CNL suppresses STAT3 phosphorylation in CLL cells and this event precedes cell death.

### Reduction in STAT3 phosphorylation is a result of CNL-induced suppression of upstream kinases including Bruton’s tyrosine kinase (BTK)

**Figure 5:**
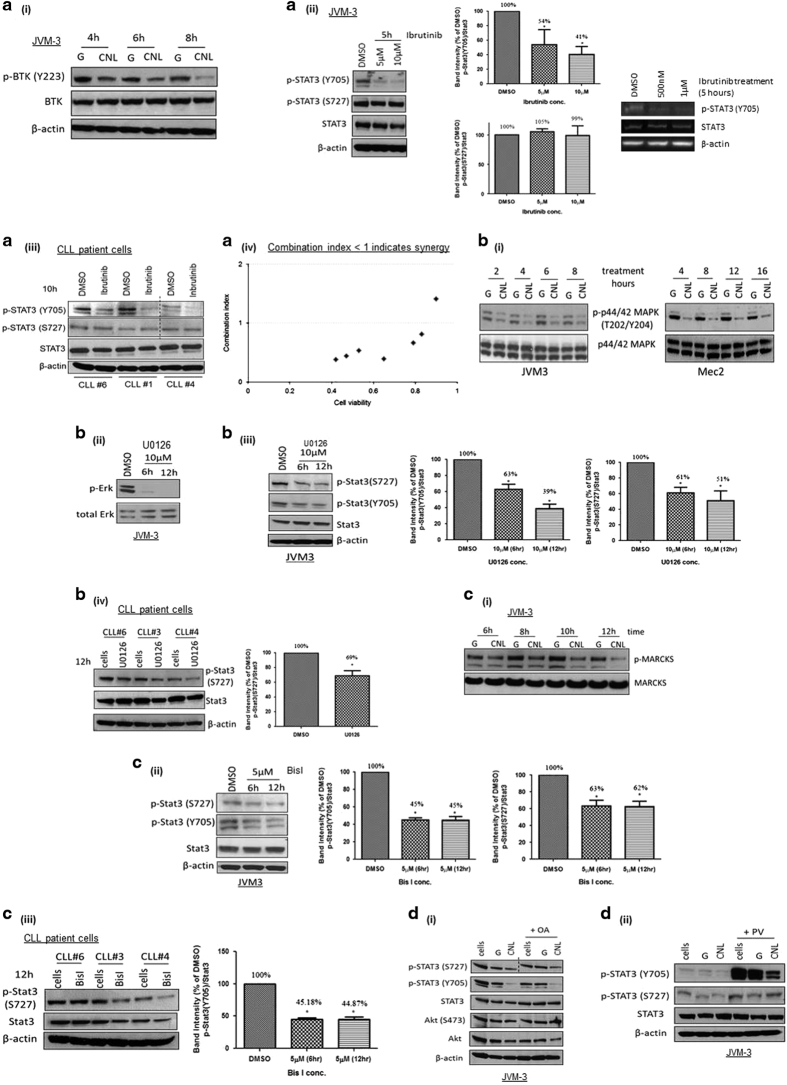
CNL suppresses STAT3 phosphorylation via multiple kinases including BTK. (**a**) (i) CNL suppresses the activity of BTK. JVM-3 cells were treated with 40 μM ghost liposomes or CNL for indicated time periods and western blotting was performed. The blots are a representative of three independent experiments. (ii) and (iii) BTK inhibitors suppress phosphorylation of STAT3 in JVM-3 cells and CLL patient cells. Cells were treated with varying concentrations of ibrutinib for indicated time periods and western blotting was performed. Graphical representation of the western blot is also shown. The blots and graphs are representative of three independent experiments or three CLL patient samples. Student’s *t*-test was used to perform statistical analysis, *P<0.05. The final western blot image was created by grouping different parts of the same film of the same gel as indicated by the black dividing line. (iv) Synergism analysis of CNL and ibrutinib treatments. JVM-3 cells were treated with single agents and co-treated with different doses of CNL (1–10 μM) and ibrutinib (1–2.5 μM) for 24 h and MTS assay was performed. The cell viability data were analyzed for synergism using Compusyn software. No synergism was observed with ghost nanoliposomes. (**b**) (i) CNL suppresses the activity of MEK1/2 kinase. JVM-3 and Mec-2 cells were treated with 40 μM ghost liposomes or CNL for indicated time periods and western blotting was performed. The blots are a representative of three independent experiments. (ii) JVM-3 cells were treated with 10 μM U0126 for indicated time periods and p-Erk levels were evaluated to confirm the effectiveness of U0126 as a MEK inhibitor. (iii) and (iv) MEK1/2 inhibitor suppresses phosphorylation of STAT3 in JVM-3 cells and CLL patient cells. Cells were treated with 10 μM U0126 for indicated time periods and western blotting was performed. The blots and graphs are representative of three independent experiments or three CLL patient samples. Student’s *t*-test was used for statistical analysis, *P<0.05. (**c**) (i) CNL suppresses the activity of PKC. JVM-3 cells were treated with 40 μM ghost liposomes or CNL for indicated time periods and western blotting was performed. The blots are a representative of three independent experiments. (ii) and (iii) PKC inhibitor suppresses phosphorylation of STAT3 in JVM-3 cells and CLL patient cells. Cells were treated with 5 μM Bis-I for indicated time periods and western blotting was performed. The blots and graphs are representative of three independent experiments or three CLL patient samples. Student’s *t*-test was used for statistical analysis, *P<0.05. (**d**) CNL does not activate phosphatases. JVM-3 cells were pretreated for 2 h with: (i) 5 nM OA; (ii) 50 μM PV, followed by 12 h of treatment with 40 μM ghost nanoliposomes or CNL. Both the inhibitors were non-toxic to cells at the specific concentration. The blots are a representative of two independent experiments. The final western blot image was created by grouping different parts of the same film of the same gel as indicated by the black dividing line.

Reduction in STAT3 phosphorylation can be a result of suppression of upstream kinases and/or activation of protein phosphatases. We first examined BTK, a tyrosine kinase critical in mediating BCR signaling in CLL cells.^30^ As shown in Figure 5a(i), we observed a significant reduction in phosphorylated BTK at Y223 in JVM-3 cells after only 4 h treatment with CNL, while total BTK remained unchanged. Phospho-Y223 is necessary of full activation of BTK, and hence is a marker of BTK activation.^31,32^ Furthermore, we also observed that treatment with varying concentrations of the BTK inhibitor ibrutinib, a drug currently prescribed for CLL, significantly reduced p-STAT3-Y705, but not p-STAT3-S727 in JVM-3 cells and three CLL patient cells (Figure 5a(ii) and (iii)).^30^ CNL did not affect levels of another tyrosine kinase, c-Abl (data not shown). Given the real-world use of ibrutinib in CLL, we sought to determine the effect of CNL and ibrutinib co-treatment on cell viability. As shown in Figure 5a(iv) and Table 2, co-treatment with CNL and ibrutinib demonstrated a synergistic reduction in cell viability. Synergism (calculated using the CompuSyn software and demonstrated by a combination index (CI) of <1) was observed across lower doses of the two drugs (5–10 μM of CNL and 1–2.5 μM of ibrutinib). A 24 h treatment with single agent ibrutinib does not affect cell viability at the doses investigated, while CNL treatment alone reduces cell viability to 70% across doses ranging from 1 to 10 μM.^13^ Ibrutinib potentiated CNL-induced reduction in cell viability as co-treatment with 10 μM CNL/2.5 μM ibrutinib further reduced cell viability to 42% (Table 2), which is a 40% enhanced effect as compared to single agent CNL treatment. This novel drug combination is interesting and warrants further investigation especially since ibrutinib is currently prescribed for CLL and CNL will be investigated in Phase 1 clinical trials for solid malignancies this year. Taken together, these results demonstrate that CNL-induced BTK inhibition mediates suppression of p-STAT3-Y705 and CNL/ibrutinib is an effective drug combination.

We also demonstrated that CNL suppresses the activity of mitogen-activated protein kinase kinase (MEK), a serine/threonine kinase. As shown in Figure 5b(i), CNL treatment significantly diminished Erk phosphorylation, a direct downstream target of MEK, within 2 h. Furthermore, treatment with U0126, a MEK inhibitor (proof of MEK inhibition shown in Figure 5b(ii)), reduced p-STAT3-S727 and p-STAT3-Y705 in both JVM-3 cells (Figure 5b(iii)) and CLL patient cells (Figure 5b(iv)). By examining the phosphorylation status of MARCKS, a direct downstream target of protein kinase C (PKC), we also observed that CNL suppresses PKC activity. CNL caused a reduction in p-MARCKS, while total MARCKS levels remained unchanged (Figure 5c(i)). Additionally, treatment with Bis-I, a PKC inhibitor also suppressed p-STAT3-S727 and p-STAT3-Y705 levels in both JVM-3 cells (Figure 5c(ii)) and CLL patient cells (Figure 5c(iii)).

We also investigated the effect of CNL on protein phosphatases. Okadaic acid (OA) is an inhibitor of serine/threonine phosphatase PP1 and PP2A. As shown in Figure 5d(i), pretreatment with OA did not rescue CNL-induced suppression of p-STAT3-S727. Pretreatment with OA rescued p-Akt-S473 after CNL treatment, confirming the effectiveness of OA as a PP2A/PP1 inhibitor. This indicates that reduction in STAT3 phosphorylation is not a result of PP1/PP2A activation. We next used pervanadate (PV) as a functional inhibitor of tyrosine phosphatases. As demonstrated in Figure 5d(ii), PV pretreatment did not rescue CNL-induced suppression of p-STAT3-Y705, indicating that this event is independent of tyrosine phosphatases activity. Basal levels of p-STAT3-Y705 increased after pretreatment with PV confirming that PV was effective in inhibiting tyrosine phosphatases. We conclude that CNL-induced suppression in STAT3 phosphorylation is not a result of protein phosphatase activity, but instead is mediated by inhibition of upstream kinases that include BTK, MEK and PKC.

### CNL suppresses the transcriptional activity of STAT3

**Figure 6:**
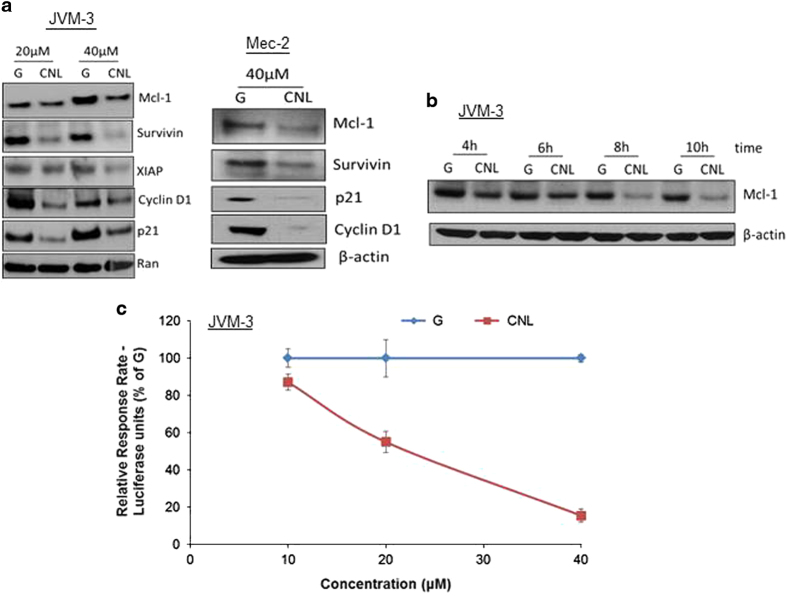
CNL suppresses the transcriptional activity of STAT3. (**a**) CNL reduces levels of STAT3-regulated genes. JVM-3 cells and Mec-2 cells were treated with 20 μM or 40 μM ghost liposomes or CNL and western blotting was performed. JVM3 cells were treated for 24 h and Mec-2 cells were treated with 48 h. The images are representative of three independent experiments. (**b**) Reduction of STAT3 phosphorylation precedes reduction of Mcl-1 levels following CNL treatment. JVM-3 cells were treated with 40 μM ghost liposomes or CNL for indicated time periods and western blotting was performed. (**c**) CNL inhibits expression of luciferase in a STAT3 luciferase reporter assay. JVM-3 cells were transfected with different luciferase constructs. Twelve hours after transfection, cells were treated with ghost nanoliposomes or CNL for 12 h and luciferase assay was performed. The graphs are representative of three independent experiments.

We sought to determine if reduction in STAT3 phosphorylation impacted transcriptional activity. CNL treatment caused a significant suppression in STAT3-regulated proteins that include, Mcl-1, survivin, XIAP, cyclin D1 and p21 (Figure 6a). CNL-induced suppression of STAT3 phosphorylation started about 3–6 h after treatment (Figure 4c(ii)) and this preceded reduction in Mcl-1, which started ~6–8 h after treatment (Figure 6b). We also confirmed these results using a luciferase reporter assay, wherein we observed a significant dose-dependent reduction in luciferase units 12 h after CNL treatment (Figure 6c). This suggests that CNL suppresses the transcriptional activity of STAT3 that leads to reduction in several pro-survival proteins.

### Overexpression of STAT3-C in CLL cells partially rescues CNL-induced cell death

**Figure 7:**
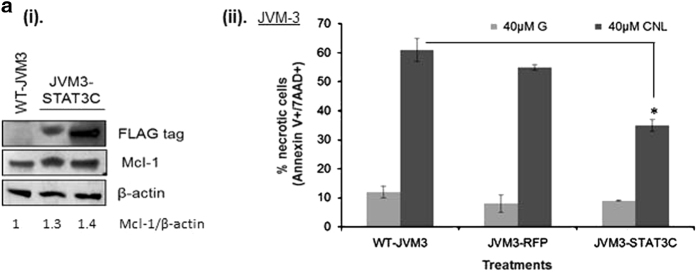
Overexpression of STAT3-C rescues CNL-induced cell death. (**a**) STAT3-C-expressing cells are resistant to CNL-induced cell death. Lentiviral transduction was performed to express STAT3-C in JVM-3 cells. Seventy-two hours after the last transduction, FACS was performed to obtain a pure population of cells expressing STAT3-C and the treatments were done. An overexpression construct expressing RFP was used as a negative control. Seventy-two hours after the last transduction cycle, cells were treated with ghost liposomes and CNL for 24 h. (i) Expression of STAT3-C was confirmed by western blotting and probing for Flag-tag. (ii) Flow cytometric analysis for Annexin-V and 7AAD was performed to quantitate % necrotic cells. Student’s *t*-test was used for statistical analysis, *P<0.05.

To confirm the role of STAT3 in CNL-induced cell death, we overexpressed an oncogenic form of STAT3 called STAT3-C. STAT3-C mimics STAT3 dimers and thus acts as constitutively active STAT3.^24^ Overexpression was performed using lentiviral transduction and cells were grown as a pure population (JVM3-STAT3C cells) after sorting using a co-expressed EGFP. STAT3-C expression was confirmed by expression of the FLAG-tag in JVM3-STAT3C cells (Figure 7a(i)). Moreover, as shown in Figure 7a(i), JVM3-STAT3C cells had a higher expression of Mcl-1, indicating higher STAT3 transcriptional activity. An overexpression vector expressing RFP was used as a control for the study (JVM3-RFP cells). Wild-type JVM-3 cells were also used as an additional control (WT-JVM3). As shown in Figure 7a(ii), WT-JVM3 and JVM3-RFP cells undergo cell death after treatment with CNL for 24 h. However, cells expressing STAT3-C were significantly more resistant to treatment with CNL compared to WT-JVM3 and JVM3-RFP cells. Since STAT3-C overexpressing cells were more resistant to CNL-induced cell death, we conclude that STAT3 partly mediates CNL-induced cell death in CLL cells.

## Discussion

In the present study, we have identified STAT3 as a molecular mediator of CNL-induced cell death in CLL. Using *TP53*^*wild-type*^ JVM-3 cells, *TP53*^*mutated*^ Mec-2 cells and primary cells from CLL patients, this study demonstrates that CNL suppresses STAT3 phosphorylation, thereby reducing levels of critical anti-apoptotic proteins, eventually inducing cell death. We are mindful that our data contrast a report in non-transformed human fibroblasts that demonstrates a crosstalk between the sphingomyelinase/ceramide pathway and the JAK/STAT signaling pathway.^33^ The authors demonstrated that exogenous ceramide or accumulation of endogenous ceramide induces STAT1 and STAT3 activation in non-transformed human fibroblasts via JAK2 kinase.^33^ However, the reader should be cognizant that there are significant variations in signal transduction pathways between non-transformed cells and malignant cells. Unlike the aforementioned report, we did not observe variations in JAK1–JAK2 phosphorylation after treatment with exogenous ceramide (data not shown). Rather, we consistently observed suppression in STAT3 activity in two CLL cell lines and patient cells. This is the first study evaluating the relation between ceramide and STAT3 in the context of malignant cells. A large body of work has delineated the signaling cascades that are targets of endogenous or exogenous ceramide to induce cancer cell death. These targets include AKT, ERK, PKCζ, survivin, phospholipase D, p38 MAPK and death receptor, to name a few.^6^ This study is the first to have demonstrated the inhibitory effect of ceramide on STAT3 signaling. Consistent with several reports in the literature, we also validate that STAT3 is a potential therapeutic target in CLL.^18,21^

Of the seven CLL patient cells tested, six responded to CNL as demonstrated by reduction in STAT3 phosphorylation and increased cell death on CNL treatment, while one patient sample was resistant to CNL treatment. We are cognizant of the limited data on primary patient samples, however, results from our previous and present work constitute proof-of-concept studies for CNL as a potential therapeutic approach for CLL.^13^ Further evidence generation for identifying CNL responders/non-responders is part of another project underway by our group. CNL is relatively non-toxic to non-malignant tissues, as shown in the extensive toxicology and stability testing in animals conducted by the Nanotechnology Characterization Laboratory (National Cancer Institute) (Detailed information on the toxicology studies of the ‘Ceramide Liposomes’ can be found at http://ncl.cancer.gov/working_technical_reports.asp). In addition, the complete toxicology packet supporting Keystone Nano’s Investigational New Drug application for CNL to FDA has been published.^34^Our current work and our group’s ongoing work in acute myeloid leukemia may lay a foundation for clinical studies investigating CNL in hematologic malignancies, as an extension to the currently underway clinical trials.^35,36^ CNL is being investigated in a phase 1 clinical trial in patients with advanced solid tumors (Investigational New Drug# 109471; Clinical trial#:NCT02834611).

Several studies have reported that STAT3 is constitutively phosphorylated on S727 and not Y705 in CLL.^17,18^ However, we observed constitutive phosphorylation at both residues in both CLL cell lines and patient cells. The studies mentioned above have not used JVM-3 or Mec-2 cells for their investigations, and our observations of dual phosphorylation may be attributed to increased activity of upstream kinases like c-Abl that contribute to STAT3 tyrosine phosphorylation as suggested by Allen *et al.*^37^ We have demonstrated that CNL significantly suppresses STAT3 phosphorylation at early time points after treatment, thereby preceding induction of cell death. We observed that CNL-induced STAT3 dephosphorylation suppresses levels of downstream pro-survival proteins like Mcl-1, survivin and XIAP that are essential for CLL cell proliferation and resistance to apoptosis, thus confirming suppression of STAT3 transcriptional activity. Results from the luciferase reporter assay confirm that CNL suppresses the transcriptional activity of STAT3 at early time points, thereby indicating that CNL-induced suppression in pro-survival proteins are effectors of cell death, rather than a consequence.

We have identified that CNL suppresses STAT3 phosphorylation at Y705 by inhibiting the activity of BTK. CNL-induced inhibition of BTK is an exciting observation since BTK is a promising target in CLL. Multiple reports have demonstrated that BTK is a critical kinase for CLL development.^38^ Ibrutinib, a BTK inhibitor, is currently used in the clinic for CLL treatment.^30^ Moreover, we have demonstrated a synergistic reduction in cell viability following combined treatment with CNL and ibrutinib. This synergism is observed at lower doses of both drugs, which is beneficial not only for the increased efficacy, but also mitigates any side effects of high doses of single agent. Though preliminary, ours is the first study providing positive evidence for this small molecule/biologic drug combination, which will be further examined in future studies. We have also demonstrated that CNL inhibits STAT3 phosphorylation at S727 via reduction in MEK and PKC kinase activity. PKC is important for CLL development and reports have signaled that PKC inhibitors may be an attractive option for CLL treatment.^39^

We performed ‘rescue’ experiments with a constitutively active dimerized form of STAT3 to definitely confirm that STAT3 is one of the mediators of CNL-induced cell death. Several publications have demonstrated that the STAT3-C protein is an oncogenic form of STAT3.^40,41^ Expression of STAT3-C also increased Mcl-1 expression, thus indicating enhanced STAT3 transcriptional activity. We observed that in contrast to control cells, cells expressing STAT3-C were more resistant to CNL-induced cell death. This proves conclusively that STAT3 partly mediates CNL-induced death in CLL cells

We have previously demonstrated that ceramide targets the Warburg effect in CLL cells.^13^ Ceramide reduced the glycolytic enzyme GAPDH, resulting in decreased glycolysis. Pretreating CLL cells with pyruvate, the end product of glycolysis, rescued CNL-induced cell death and CNL-induced ATP depletion. Thus, targeting GAPDH is one mechanism by which CNL inhibits aerobic glycolysis.^13^ In the present study, we have demonstrated that CNL suppresses STAT3 phosphorylation, resulting in subsequent inhibition of STAT3 transcriptional activity. Our preliminary studies have demonstrated that GAPDH expression may be partially regulated by the transcriptional activity of STAT3 (data not shown), which establishes a potential link between CNL-induced suppression in STAT3 phosphorylation and CNL-induced inhibition of Warburg effect. Another possible link may be established on the basis of evidence provided by Demaria *et al.*^42^ They have shown that STAT3 acts as a master regulator of cell metabolism by activating HIF-1α-dependent aerobic glycolysis.^42^ Thus, we speculate that CNL-induced suppression in STAT3 phosphorylation might suppress HIF-1α expression and subsequently suppress HIF-1α-dependent aerobic glycolysis. This is another mechanism by which CNL-induced inhibition in STAT3 phosphorylation may be linked to suppression in the Warburg effect, a hypothesis to be tested in our future work.

In addition to the oncogenic function of constitutive p-STAT3-Y705 as a transcription factor, an extranuclear pro-oncogenic role of constitutive p-STAT3-S727 was uncovered in the past few years. It was reported that STAT3 associates with complex I and II of the electron transport chain and is required for optimal mitochondrial respiration.^43^ Additionally, phosphorylation at S727 of mitochondrial STAT3 was identified to be essential for its mitochondrial function and for Ras-dependent oncogenic transformation.^44^ Following this revelation, reports have documented the pivotal role of mitochondria-associated constitutive p-STAT3-S727 in pathogenesis of breast cancer, pancreatic cancer, murine myeloproliferative neoplasms and also CLL.^19,45–47^ Having established the ability of CNL to suppress p-STAT3-S727 in CLL, it will be interesting to look at the effect on phosphorylation of mitochondrial STAT3, effect on electron transport chain and overall mitochondrial respiration in CLL.

In conclusion, this work is the first body of evidence demonstrating that CNL suppresses STAT3 phosphorylation in cancer cells and that STAT3 is a mediator of CNL-induced cell death. Also, we provide the first evidence for CNL-induced suppression of BTK activity and synergistic cell death by CNL/ibrutinib combination. Our findings are clinically relevant since CNL is currently being investigated in a first-in-human phase I 3+3 dose escalation clinical trial in solid tumors (NCT02834611) at the University of Maryland, University of Virginia and Medical University of South Carolina.^34^ This work thus opens up a wide avenue of research directed toward examining the ceramide-STAT3 drug-target combination in other cancer models. We believe that combination therapies with CNL and STAT3 inhibitors should be explored in STAT3-dependent cancers.

## Figures and Tables

**Table 1:** Patient characteristics

*CLL patient*	*Gender*	*Age*	*Disease stage (Rai stage)*
1	Male	71	I
2	Male	65	0
3	Male	79	0
4	Female	83	0
5	Female	55	0
6	Female	53	0
7	Female	67	I

**Table 2:** Synergism analysis for CNL and ibrutinib co-treatment in JVM-3 cells

*CNL dose*	*Ibrutinib dose*	*Cell viability*	*Combination index*	*Effect*
5 μM	1 μM	83%	0.81	Synergism
5 μM	2.5 μM	79%	0.66	Synergism
10 μM	1 μM	53%	0.53	Synergism
10 μM	2.5 μM	42%	0.38	Synergism

Treatment with ibrutinib alone at 1 and 2.5 μM for 24 h did not affect cell viability. Twenty-four hours treatment with CNL at 5 and 10 μM reduced cell viability to 90% and 70%, respectively.
